# CT mimics of peritoneal carcinomatosis

**DOI:** 10.4103/0971-3026.59757

**Published:** 2010-02

**Authors:** S Smiti, KV Rajagopal

**Affiliations:** Department of Radio-diagnosis and Imaging, Kasturba Medical College, Manipal, Karnataka, India

**Keywords:** Carcinomatosis, neoplastic, peritoneal

## Abstract

Peritoneal carcinomatosis is a term used to describe widespread metastases of cancerous tumors in the peritoneal cavity. It is most common in carcinomas of the gastrointestinal tract (GIT) and ovaries, and must be considered to be the main diagnosis even when the primary is not known. A wide variety of disease processes mimic peritoneal carcinomatosis. Precise diagnosis based on imaging alone is often difficult and very often the final diagnosis is only obtained after appropriate histopathology or microbiology.

## Introduction

Peritoneal carcinomatosis is a metastatic manifestation of many organ-based malignancies, particularly carcinomas of the gastrointestinal tract (GIT) and ovaries, and must be considered as the first possibility even in the absence of a known primary. There are several neoplastic and non-neoplastic conditions that may mimic peritoneal carcinomatosis on CT scan. These include lymphomas, gastrointestinal stromal tumors (GIST), granulomatous infections like tuberculosis, and primary peritoneal malignancies such as mesotheliomas.

## Discussion

### Peritoneal carcinomatosis

**Figure 1:**
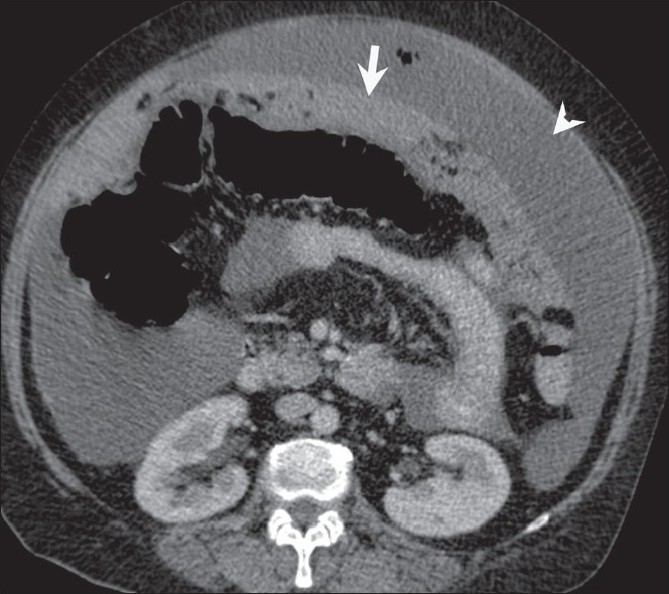
Peritoneal carcinomatosis. Omental caking (arrow) and ascites (arrowhead) are seen on an axial, contrast-enhanced CT scan of the abdomen, in a 55-year-old woman, a known case of carcinoma ovary with raised CA-125 levels

Peritoneal carcinomatosis without distant metastases represents locoregional disease and calls for aggressive locoregional treatment. Most CT scan findings are however nonspecific as both neoplastic and non-neoplastic pathologies of the peritoneum present as soft-tissue masses, with or without ascites.[1] In addition, there may also be a cystic component, necrosis, calcification, or significant contrast enhancement. Sometimes, peritoneal nodules can simulate unopacified bowel loops and hence adequate bowel opacification is important for accurate diagnosis.[2] The CT appearance of neoplastic infiltration of the greater omentum can range from increased density of fat anterior to the colon or small bowel, to large masses, called omental cakes, separating the colon and small bowel from the anterior abdominal wall,[Figure 1].

**Figure 2 (a,b):**
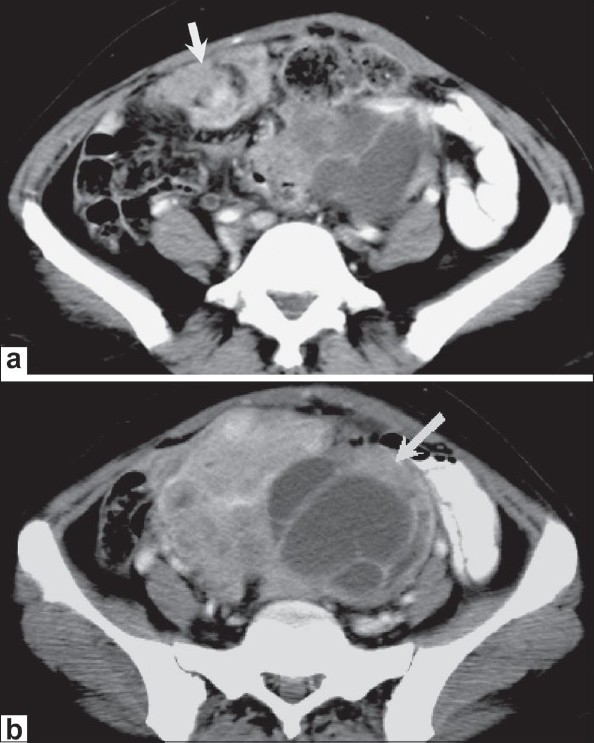
Peritoneal carcinomatosis. Axial contrast-enhanced CT scan of the mid-pelvis (a) shows a heterogeneously enhancing peritoneal/omental deposit (arrow). An axial image (b), a little inferior to A shows a large heterogeneous mass (arrow) in the pelvis with solid and cystic components. Biopsy showed papillary adenocarcinoma of the ovary

Very often though, the diagnosis is relatively easy when associated ovarian [Figure 2a and 2b] or gastric neoplastic disease is seen. In the absence of a primary neoplasm and sometimes even in the presence of ovarian or gastric and bowel masses, other disease entities such as GIT lymphomas, GIST of the omentum and mesentery, peritoneal tuberculosis, and primary neoplasms of the peritoneum like primary peritoneal mesothelioma, can all mimic peritoneal carcinomatosis.

This pictorial essay is based on our experience with patients with CT features that mimicked peritoneal carcinomatosis; in all cases, the diagnosis was confirmed on histopathology.

### Lymphoma

**Figure 3 (a-c):**
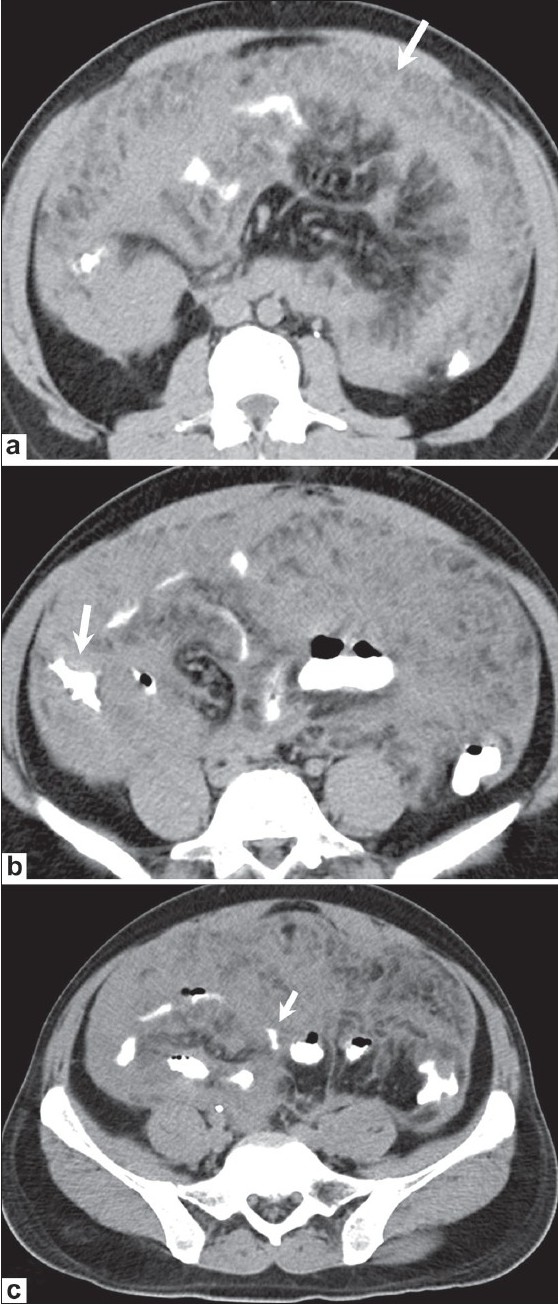
Lymphoma. A 36-year-old male presented with abdominal pain. Axial, contrast-enhanced CT scans of the abdomen show diffuse omental thickening (arrow in a) with bowel wall thickening in the ascending colon (arrow in b) and small bowel (arrow in c). Biopsy was consistent with small-cell, cleaved non-Hodgkin lymphoma

**Figure 4:**
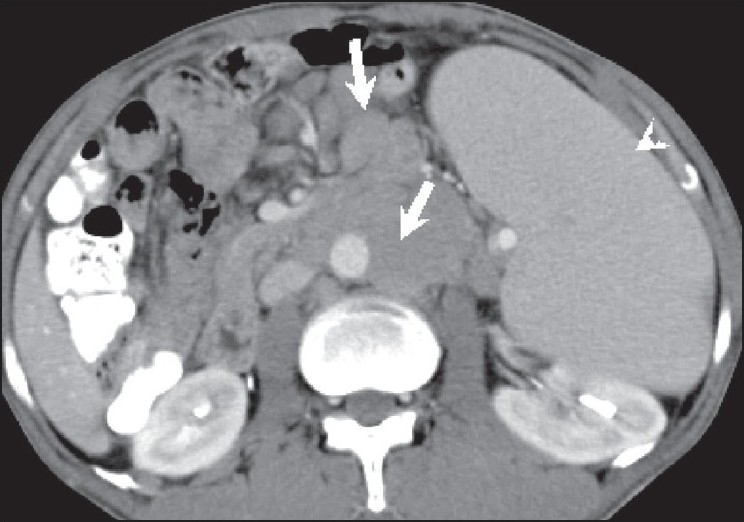
Lymphoma. A 45-year-old male with non-Hodgkin lymphoma has para-aortic and mesenteric lymphadenopathy (arrows) along with splenomegaly (arrowhead), on a contrast-enhanced, axial CT scan of the abdomen

**Figure 5 (a,b):**
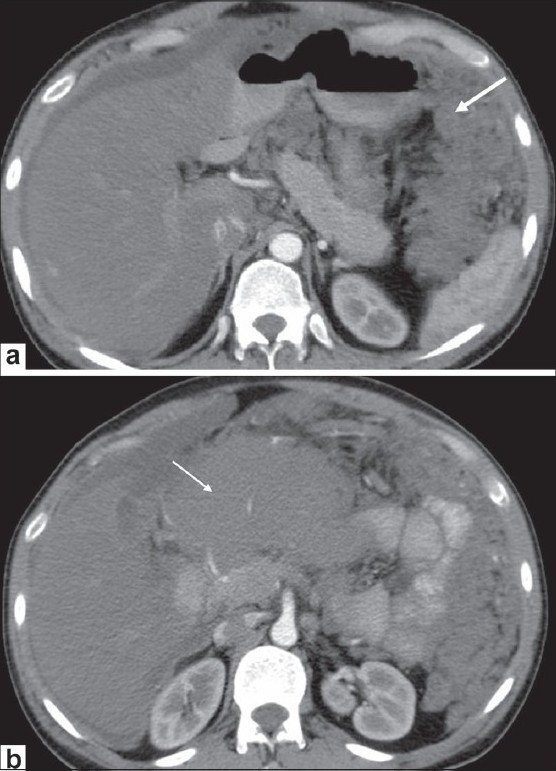
Lymphoma. A 34-year-man with 1-month history of abdominal pain has omental thickening (arrow in a) and an epigastric mass (arrow in b), on contrast-enhanced, axial CT scans of the abdomen. Histopathology was suggestive of non-Hodgkin lymphoma

Peritoneal lymphomatosis due to GIT lymphoma may be seen on CT as omental caking or masses, with diffuse peritoneal thickening [Figure 3a] or ascites.[3] Associated findings that may help in distinguishing lymphoma from peritoneal carcinomatosis include aneurysmal dilatation of a bowel segment, with a thickened wall [Figure 3b and c] and splenic enlargement[4] [Figure 4]. The classic appearance on CT is of confluent masses causing encasement of the superior mesenteric artery and vein, producing a ‘sandwich sign’;[5] these masses are bulky, soft, non-obstructing neoplasms [Figure 5a and b], with a tendency to be less vascular than carcinomas. There is usually homogenous attenuation, without significant necrosis with marked bowel wall thickening. The differential diagnosis of mesenteric lymphadenopathy also includes metastases and reactive lymphadenopathy due to granulomatous infections, Crohn disease, etc.[2] Splenic involvement and large non-necrotic masses and lymph nodes help make this diagnosis.

### Primary peritoneal mesothelioma

**Figure 6 (a,b):**
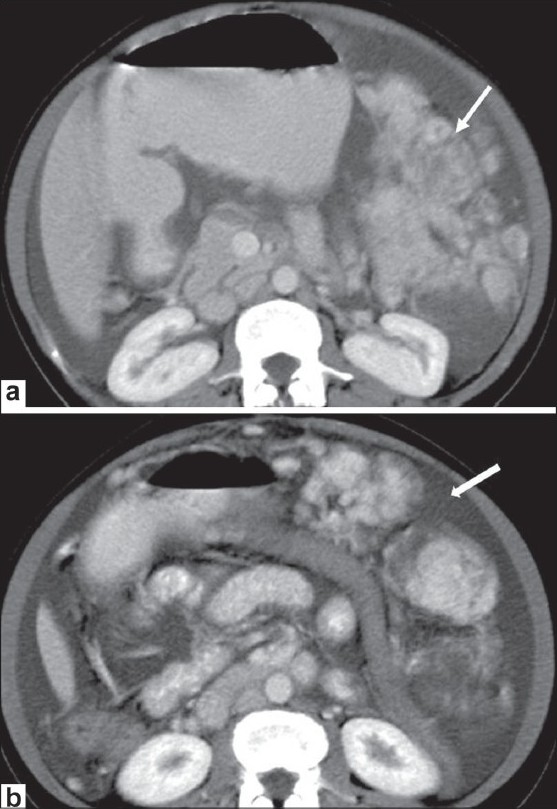
Peritoneal mesothelioma. A 60-year-old woman presented with abdominal distension and loss of appetite. Axial, contrastenhanced images of the abdomen show a lobulated heterogenously enhancing intraperitoneal lesion in the left hemi-abdomen (arrow in a) with ascites (arrow in b); however, no calcification was seen within the mass. Biopsy was suggestive of primary peritoneal mesothelioma

Malignant primary peritoneal mesothelioma, though rare, can be seen as a large confluent mass [Figure 6a], which may be nodular or diffuse, with or without ascites [Figure 6b]. Calcification is uncommon.[1] Approximately 30% arise primarily from the peritoneum, with the rest arising from the pleural surface. It can cause scalloping of, or a mass effect on, adjacent abdominal organs. A history of exposure to asbestos is found in a few cases. Unlike in pleural mesothelioma, associated calcified peritoneal plaques are uncommon. High-power microscopy may show hyperchromatic nuclei and large cells with peripheral dense cytoplasm.

### Gastrointestinal stromal tumors

GIST refers to tumors arising from the mesenchymal tissue of the GIT; they commonly possess spindle cells and show c-kit protein positivity.[6] Although c-kit expression may be seen in other malignant tumors, it has a high specificity for GIST. GIST is often solitary and arises most commonly from the stomach (60-70%), followed by small bowel (20-25%) and, rarely, the rectum (5%), esophagus, colon, and appendix.[3] GIST is rarely seen arising from the mesentery, omentum, and retroperitoneum and is usually large in size at the time of presentation. It may sometimes be an incidental finding owing to the submucosal origin of the tumor and exophytic nature of the tumor growth.[7]

**Figure 7 (a,b):**
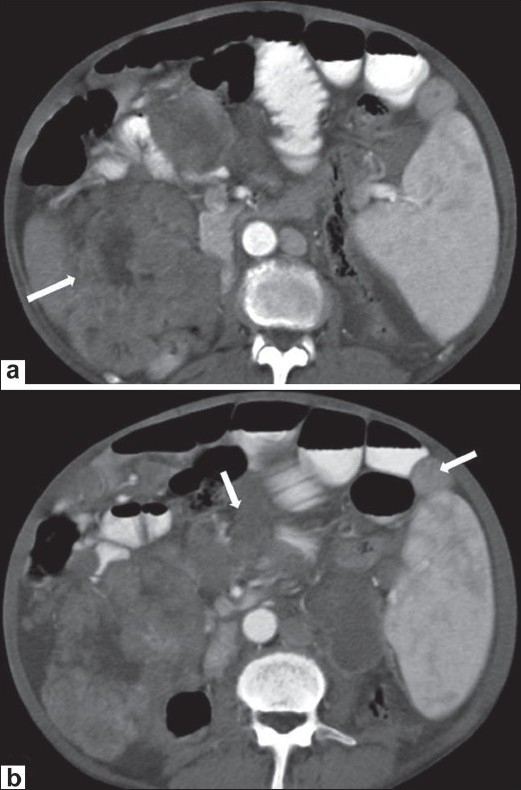
GIST. Axial, contrast-enhanced CT scan of the abdomen in a 65-year-old male with diffuse abdominal pain shows a large, heterogenously enhancing intraperitoneal mass having central necrosis (arrow in a) with multiple peritoneal deposits (arrow in b). This was confirmed on histopathology

GIST of the omentum and mesentery may present with diffuse peritoneal seeding, mimicking mesenteric carcinomatosis. In a study by Kim *et al*., primary GIST in the omentum and mesentery were seen as well-circumscribed, large masses containing areas of hemorrhage, necrosis [Figure 7a], or cystic degeneration.[8] Peritoneal deposits may also be seen [Figure 7b]. One differentiating feature of GIST is their hypervascularity because of which, even if central necrosis or cystic degeneration is present, there may be peripheral enhancement with surrounding dilated vessels.[9]

### Peritoneal tuberculosis

**Figure 8:**
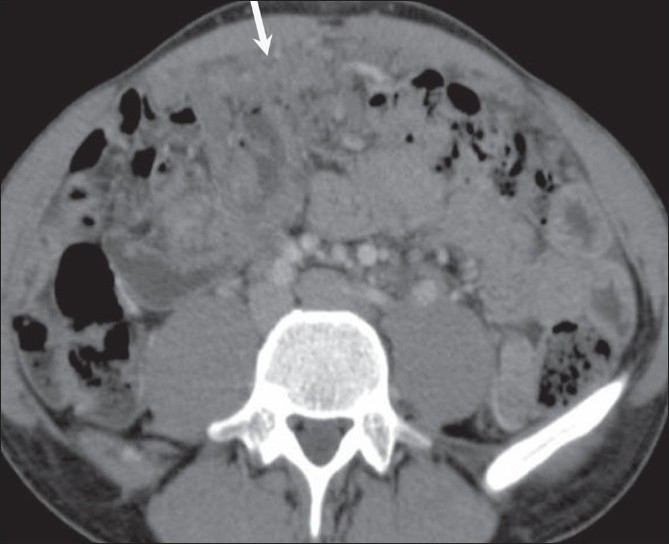
Tuberculosis. Axial, contrast-enhanced CT scan in a 35-year-old man with abdominal tuberculosis shows diffuse omental thickening (arrow)

**Figure 9:**
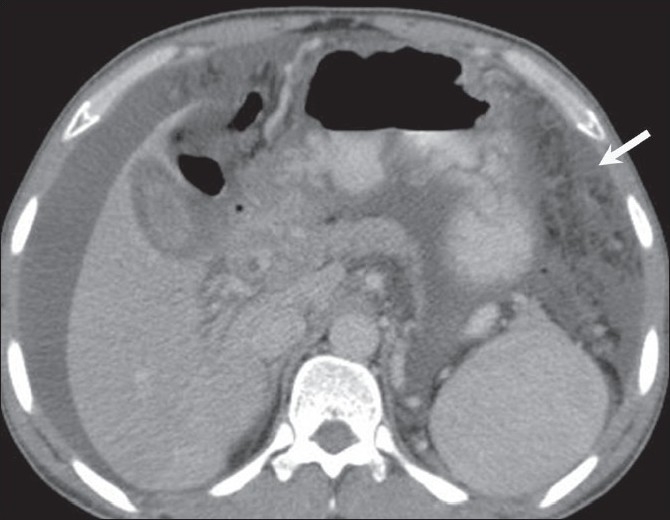
Tuberculosis. Axial, contrast-enhanced CT scan in a 40- year- old woman with abdominal pain shows omental thickening (arrow) along with ascites. A possibility of peritoneal carcinomatosis was considered. Histopathology was suggestive of tuberculosis

Peritoneal tuberculosis, in particular, can be a difficult and elusive diagnosis to make and may mimic metastases from ovarian cancer and other nontuberculous granulomatous diseases because of the vague symptoms and nonspecific radiographic, pathologic, and laboratory findings. Tuberculous peritonitis may be of wet, fixed fibrotic, and dry plastic types.[10] The wet type presents as free or loculated ascites with septae. The fixed fibrotic type may present as an omental and mesenteric mass, with matted bowel loops, and the dry plastic type can show thickened peritoneum and necrotic lymph nodes, though there is often an overlap between these two types.[2] A high index of suspicion for peritoneal tuberculosis is important if unnecessary elaborate surgery and delay in treatment are to be avoided.[11] The CT scan findings include omental cake-like masses [Figure 8], nodules,[10] and a smudge pattern. The peritoneal thickening is usually smooth as compared to the nodularity seen in peritoneal carcinomatosis.[12] Peritoneal tuberculosis can mimic peritoneal carcinomatosis [Figure 9]. A few cases of abdominal tuberculosis may even show elevation of CA 125. The presence of necrotic mesenteric and retroperitoneal lymph nodes, especially in younger patients helps clinch this diagnosis.

Other lesions such as papillary serous carcinoma, desmoplastic small round-cell tumor, and mesenchymal tumors, including both benign and malignant tumors may occur but are difficult to diagnosis on imaging findings alone.

CT scan plays an important role in the detection of peritoneal carcinomatosis and its mimics. However, the exact diagnosis and characterization of lesions may be difficult due to the overlap of imaging findings. CT scan can also play an important role in guiding biopsy for tissue diagnosis and can provide the surgeon with a ‘road map’ prior to cytoreductive surgery. Since a precise diagnosis based on imaging findings alone is often not possible, histopathology is mandatory to confirm the diagnosis.
